# Impact of Neoadjuvant Chemoradiation on Pathologic Response in Patients With Localized Pancreatic Cancer

**DOI:** 10.3389/fonc.2020.00460

**Published:** 2020-04-15

**Authors:** David Wittmann, William A. Hall, Kathleen K. Christians, Chad A. Barnes, Neil R. Jariwalla, Mohammed Aldakkak, Callisia N. Clarke, Ben George, Paul S. Ritch, Matthew Riese, Abdul H. Khan, Naveen Kulkarni, John Evans, Beth A. Erickson, Douglas B. Evans, Susan Tsai

**Affiliations:** ^1^The LaBahn Pancreatic Cancer Program, Department of Surgery, Medical College of Wisconsin, Milwaukee, WI, United States; ^2^The LaBahn Pancreatic Cancer Program, Department of Radiation Oncology, Medical College of Wisconsin, Milwaukee, WI, United States; ^3^The LaBahn Pancreatic Cancer Program, Department of Medicine, Division of Hematology Oncology, Medical College of Wisconsin, Milwaukee, WI, United States; ^4^The LaBahn Pancreatic Cancer Program, Division of Gastroenterology, Medical College of Wisconsin, Milwaukee, WI, United States; ^5^The LaBahn Pancreatic Cancer Program, Department of Radiology, Medical College of Wisconsin, Milwaukee, WI, United States; ^6^The LaBahn Pancreatic Cancer Program, Department of Pathology, Medical College of Wisconsin, Milwaukee, WI, United States

**Keywords:** pancreatic cancer, neoadjuvant therapy, histologic response, lymph nodes, radiation

## Abstract

**Introduction/Background:** Multimodal neoadjuvant therapy has resulted in increased rates of histologic response in pancreatic tumors and adjacent lymph nodes. The biologic significance of the collective response in the primary tumor and lymph nodes is not understood.

**Methods:** Patients with localized PC who received neoadjuvant therapy and surgery with histologic assessment of the primary tumor and local-regional lymph nodes were included. Histopathologic response was classified using the modified Ryan score as follows: no viable cancer cells (CR), rare groups of cancer cells (nCR), residual cancer with evident tumor regression (PR), and extensive residual cancer with no evident tumor regression (NR). Nodal status was defined by number of lymph nodes (LN) with tumor metastases: N0 (0 LN), N1 (1–3), N2 (≥4).

**Results:** Of 341 patients with localized PC who received neoadjuvant therapy and surgery, 107 (31%) received chemoradiation alone, 44 (13%) received chemotherapy alone, and 190 (56%) received chemotherapy and chemoradiation. Histopathologic response consisted of 15 (4%) CRs, 59 (17%) nCRs, 188 (55%) PRs, and 79 (23%) NRs. Patients who received chemotherapy alone had the worst responses (*n* = 21 for NR, 48%) as compared to patients who received chemoradiation alone (*n* = 25 for NR, 24%) or patients who received both therapies (*n* = 33 for NR, 17%) (Table 1; *p* = 0.001). Median overall survival for all 341 patients was 39 months; OS by histopathologic subtype was not reached (CR), 49 months (nCR), 38 months (PR), and 34 months (NR), respectively (*p* = 0.004). Of the 341 patients, 208 (61%) had N0 disease, 97 (28%) had N1 disease, and 36 (11%) had N2 disease. In an adjusted hazards model, modified Ryan score of PR or NR (HR: 1.71; 95% CI: 1.15–2.54; *p* = 0.008) and N1 (HR: 1.42; 95% CI: 1.1.02–2.01; *p* = 0.04), or N2 disease (HR: 2.54, 95% CI: 1.64–3.93; *p* < 0.001) were associated with increased risk of death.

**Conclusions:** Neoadjuvant chemotherapy alone is associated with lower rates of pathologic response. Patients with CR or nCR have a significantly improved OS as compared to patients with PR or NR. Nodal status is the most important pathologic prognostic factor. Neoadjuvant chemoradiation may be an important driver of pathologic response.

## Introduction

In the past decade, there has been significant progress in the treatment sequencing of patients with localized pancreatic cancer (PC) (1). Neoadjuvant therapy has become the standard treatment for borderline resectable (BLR) PC and has been adopted at multiple centers for the management of resectable disease as well (2, 3). Although neoadjuvant therapy has been increasingly accepted as the preferred treatment paradigm for patients with localized PC, there remains a lack of consensus regarding the optimal regimen and treatment duration. For example, there is considerable heterogeneity with respect to the duration of chemotherapy and the inclusion of chemoradiation. Given the high rate of metastatic disease recurrence following pancreatectomy, early delivery of systemic chemotherapy may help to control clinically occult micrometastatic disease which is present in most patients at the time of diagnosis (4). As systemic disease control improves, locoregional recurrence in patients with PC may become even more problematic; at present, up to 50% of patients have a positive resection (R1) margin following upfront surgical resection and isolated local recurrence occurs in a minimum of 24–50% of patients who undergo potentially curative surgery (5–7). Currently, no level one evidence exists to support a preferred neoadjuvant treatment program. The results of the ongoing Alliance (A021501) trial for patients with BLR PC in which patients are randomized to receive neoadjuvant chemotherapy with or without stereotactic body radiation are eagerly anticipated (NCT02839343).

Assessment of histopathologic response may provide direct evidence of treatment effect on the primary tumor and regional lymph nodes and may be a surrogate marker for disease control in radiographically occult distant metastatic sites. In other solid tumors, robust histologic response to neoadjuvant therapy (complete response [CR]) has been associated with improved overall survival and decreased rates of local recurrence (8–10). Unfortunately, in patients with operable PC, CR occurs in only 3–11% of patients following neoadjuvant therapy (11–13). Information on the prognostic importance of non-CR histologic responses to neoadjuvant therapy in the primary tumor and local-regional lymph nodes is limited, as is the understanding of how the components of neoadjuvant therapy (chemotherapy vs. chemoradiation) impact pathologic response. The purpose of this study was to compare the histopathologic response at the primary tumor, lymph node status, and associated overall survival (OS) in patients with localized pancreatic cancer who received neoadjuvant therapy consisting of chemotherapy, chemoradiation, or chemotherapy followed by chemoradiation.

## Methods

### Study Subjects

This was a retrospective analysis of a prospectively maintained database which included consecutive patients with resectable and BLR PC who received neoadjuvant therapy and surgery for pancreatic adenocarcinoma at a single academic institution from 2009 to 2018. Clinical stage at the time of diagnosis was determined using objective radiographic criteria based on computed tomography (CT) imaging, as previously described (14, 15). This study was approved by the Institutional Review Board of the Medical College of Wisconsin.

### Neoadjuvant Therapy

In general, neoadjuvant therapy for patients with resectable PC consisted of 50.4 Gy of radiation delivered over 28 fractions with concurrent gemcitabine or capecitabine. Regional nodal radiation was routinely included and typically involved the proximal celiac and superior mesenteric (artery and vein) vessels, along with the splenic or porta hepatis nodes depending on primary tumor location. A subset of patients with resectable PC received 2 months of chemotherapy on a clinical trial utilizing molecular profiling (16). For patients with BLR PC, neoadjuvant therapy consisted of a minimum of 2 months of chemotherapy followed by chemoradiation (50.4 Gy over 28 fractions). Staging with CT imaging and laboratory studies (carbohydrate antigen 19-9 [CA19-9] and carcinoembryonic antigen) occurred at diagnosis (pretreatment), after completion of neoadjuvant therapy (preoperative), and after surgery (post-treatment); more frequent re-staging occurred in patients who received a lengthier course of neoadjuvant therapy. Pancreatectomies were performed in a standard fashion as previously described (17, 18).

### Histopathologic Response Classification

Histopathologic response was abstracted from synoptic pathology reports using the College of American Pathologists Protocol for the Examination of Specimens from Patients with Carcinoma of the Exocrine Pancreas (Pancreas Exocrine 4.0.0.1) (19). Treatment effect was classified using the College of American Pathologist Modified Ryan Scheme for Tumor Regression Score according to the following criteria: 0-complete response (CR), if no viable cancer cells were identified in the primary tumor or lymph node; 1-near complete response (nCR), if single cells or rare small groups of cancer cells were in the primary tumor; 2-partial response (PR), if residual cancer was present in the primary tumor with evident tumor regression, but more than single cells or rare small groups of cancer cells; 3-poor or no response (NR), if extensive residual cancer was present in the primary tumor with no evident tumor regression (20).

### Nodal Status

Nodal status was abstracted from the synoptic pathology reports and was defined according to Regional Lymph Node (N) Categories, established in the 8th edition of the AJCC staging manual. The following criteria were utilized: N0, no regional lymph node involvement; N1, 1–3 regional lymph nodes containing cancer; N2, 4 or more regional lymph nodes containing cancer (21).

### Statistical Analysis

Categorical variables were compared using the Fischer's Exact test. All continuous variables were analyzed using the Mann-Whitney U test. Overall survival (OS) was calculated from the time of tissue diagnosis to the date of death or last follow-up. Deaths from any cause were included in the survival analysis. OS was estimated using the Kaplan-Meier method as well as log-rank test. Multivariable analysis via the Cox proportional-hazards model was used to examine the effect of prognostic factors on overall survival. All statistical analyses were performed using Stata 15.1 (StataCorp, College Station, Texas).

## Results

From 2009 to 2018, 517 patients with resectable (*n* = 216, 42%) or BLR (*n* = 301, 58%) PC were treated with neoadjuvant therapy. All intended neoadjuvant therapy and surgery was completed in 361 (70%) of the 517 patients; 169 (78%) of the 216 resectable and 192 (53%) of the 301 BLR patients. Of the 361 patients, a total of 20 (6%) were excluded from the analysis due to receipt of an investigational drug during neoadjuvant therapy (*n* = 1), postoperative death within 90 days (*n* = 4), lack of follow-up after surgery (*n* = 3), postoperative pathology demonstrating that metastatic disease was present at the time of surgery (*n* = 2), or a missing modified Ryan score (*n* = 10). A total of 341 patients, who both completed all intended neoadjuvant therapy and surgery and had a modified Ryan score were included in this analysis.

Patients were categorized based on the neoadjuvant treatment received (Table 1); 107 (31%) patients received chemoradiation (chemoXRT), 44 (13%) received chemotherapy alone (chemo alone), and 190 (56%) received both chemotherapy and chemoradiation (both). Treatment sequencing was highly correlated with clinical stage; 138 (88%) of the 161 patients with resectable PC received either chemoXRT or chemo alone, while 170 (89%) of the 181 patients with BLR PC received both chemotherapy and chemoXRT (*p* < 0.001). Of the 234 patients who received chemotherapy alone or chemotherapy and chemoradiation, the most common regimen was FOLFIRINOX (*n* = 116, 49%), gemcitabine/nab-paclitaxel (*n* = 43, 18%), 5-FU based doublet (*n* = 33, 13%), gemcitabine-based triplet (*n* = 4, 2%), alternative gemcitabine-based doublet (*n* = 27, 11%), or single agent gemcitabine (*n* = 10, 4%). Chemoradiation consisted of 50.4 Gy of radiation delivered over 28 fractions with concurrent gemcitabine or capecitabine.

**Table 1:** Clinical characteristics by neoadjuvant treatment modality.

**Characteristic**	**Total *n* = 341**	**ChemoXRT *n* = 107**	**Chemotherapy alone *n* = 44**	**Both *n* = 190**	***p*-value**
**Clinical stage**, ***n*** **(%)**
Resectable	161 (47)	103 (96)	38 (86)	20 (10)	<0.001
BLR	181 (53)	4 (4)	6 (14)	170 (89)	
Age in years, median (IQR)	65 (12)	67 (14)	64 (12)	65 (13)	0.07
Gender (Female), *n* (%)	169 (50)	59 (55)	24 (55)	85 (45)	0.19
Charlson Comorbidity Index, median (IQR)	3 (2)	3 (2)	2 (1)	3 (2)	0.05
Body Mass Index, median (IQR)	27 (7)	27 (7)	27 (6)	27 (7)	0.88
Tumor size (cm), median (IQR)	2.8 (2)	2.3 (1.3)	2.2 (1.1)	3 (1.3)	<0.001
CA19-9 at Diagnosis, U/mL, median (IQR)¥	188 (497)	109 (293)	216 (753)	246 (616)	0.13
Preoperative CA19-9, U/mL, median (IQR)¥	33 (59)	34 (61)	49 (140)	28 (50)	0.32
Postoperative CA19-9, U/mL, median (IQR)¥	17 (28)	18 (22)	15 (27)	16 (30)	0.97
Elevated Preoperative CA19-9, n (%)	138 (46)	47 (48)	23 (55)	68 (42)	0.31
Elevated Postoperative CA19-9, n (%)	20 (38)	29 (55)	19 (24)	19 (40)	0.18
**Histopathologic response**, ***n*** **(%)**
CR	15 (4)	4 (4)	1 (2)	10 (5)	0.001
nCR	59 (17)	13 (12)	8 (18)	38 (20)	
PR	188 (55)	65 (61)	14 (32)	109 (58)	
NR	79 (23)	25 (24)	21 (48)	33 (17)	
**Pathological T stage**, ***n*** **(%)**
T0	15 (4)	4 (4)	1 (2)	10 (5)	0.91
T1	107 (32)	37 (35)	15 (34)	55 (29)	
T2	173 (51)	52 (49)	24 (55)	97 (51)	
T3	46 (13)	14 (13)	4 (9)	28 (15)	
Median Pathologic Tumor Size, (cm)	2.5 (1.6)	2.5 (1.7)	2.5 (1.2)	2.5 (1.5)	0.86
**Node status**, ***n*** **(%)**
N0	208 (61)	65 (61)	19 (43)	124 (65)	0.06
N1	97 (28)	28 (26)	18 (41)	51 (27)	
N2	36 (11)	14 (13)	7 (16)	15 (8)	
**Margin status**, ***n*** **(%)**
Negative	295 (87)	93 (87)	38 (86)	164 (86)	0.99
Positive	46 (13)	14 (13)	6 (14)	26 (14)	

### Histopathologic Response of the Primary Tumor

The median tumor size as measured on final pathology after induction therapy was 2.8 cm (IQR: 2); 2.3 cm (IQR: 1.3) for patients who received chemoradiation, 2.2 cm (1.0) for patients who received chemotherapy alone, and 3.0 cm (1.3) for patients who received both therapies (*p* < 0.001). The histologic response by type of neoadjuvant treatment is summarized in Table 1. Of the 341 patients, 15 (4%) had a CR, 59 (17%) had a nCR, 188 (55%) had a PR, and 79 (23%) had NR. Of the 15 patients with a CR in the primary tumor, four patients were treated with chemoXRT, one received chemo alone, and 10 patients received both therapies (*p* = 0.65). Rates of CR and nCR were similar between the treatment groups, however, 48% (21/44) of patients who received chemo alone had NR as compared to 23% (*n* = 25/107) of patients who received chemoXRT and 17% (*n* = 33/190) of patients who received both therapies (*p* = 0.001). The majority of patients treated with either chemoXRT (*n* = 65/107, 61%) or chemotherapy and chemoXRT (*n* = 109/190, 57%) had a PR in the primary tumor. The median tumor size at diagnosis, as measured by CT scan, was not associated with pathologic response following neoadjuvant therapy; the median size of tumors in patients who had CR, nCR, PR, and NR was 3.5, 3.0, 2.9, and 2.8, respectively.

**Figure 1:**
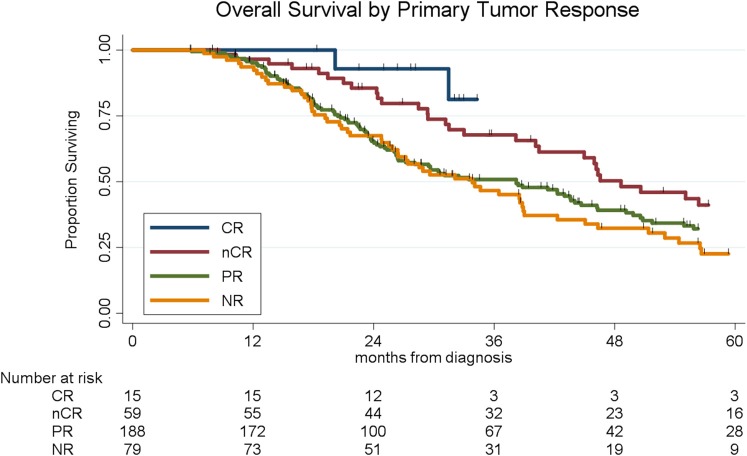
Overall survival by histopathologic response of the primary tumor.

**Figure 2:**
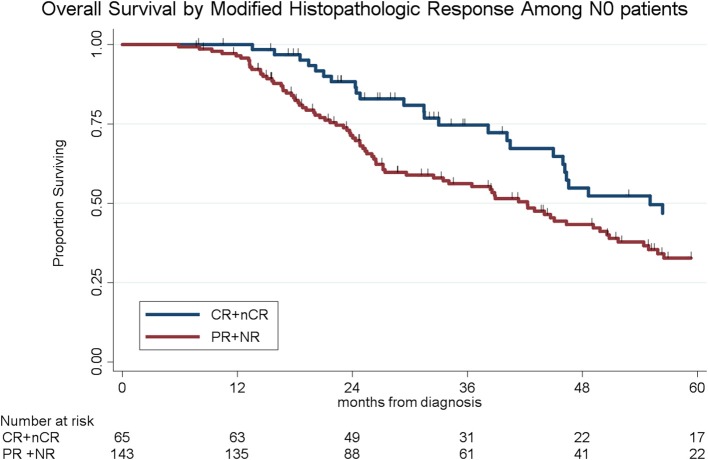
Overall survival by modified histopathologic response of the primary tumor.

Histopathologic response was correlated with OS. Of the 341 patients, the median OS was 39 months for the entire cohort; OS by histopathologic subtype was not reached (CR), 49 months (nCR), 38 months (PR), and 34 months (NR), respectively (log-rank *p* = 0.004, Figure 1). There was no significant difference in OS among patients with CR and nCR or among patients with a PR and NR. We therefore simplified the histological classification into two groups (CR/nCR vs. PR/NR); median OS for those with a CR/nCR was 51 months vs. 34 months for those with a PR/NR (log-rank *p* = 0.002, Figure 2). In a multivariable logistic regression model, combination chemotherapy and chemoXRT was associated with an increased odds of having a CR/nCR (OR: 1.67; 95%CI: 1.23–3.24, *p* = 0.03), while male gender was associated with a decreased odds of CR/nCR (OR: 0.53; 95% CI: 0.30–0.95, *p* = 0.02).

### Pathologic Lymph Node Status

**Figure 3:**
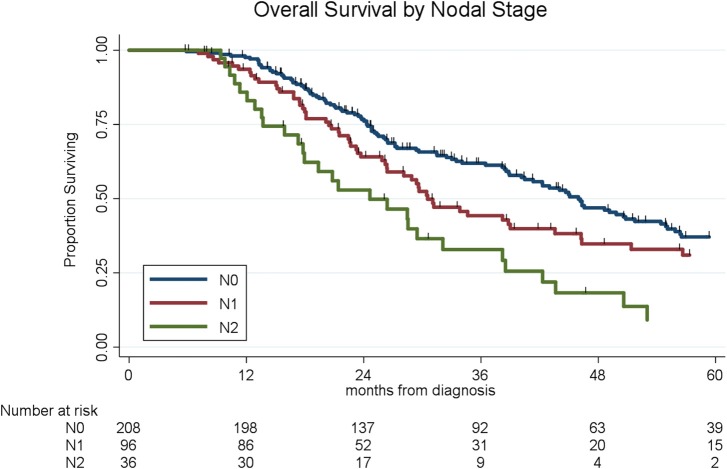
Overall survival by lymph node status.

Lymph node metastases were present in 133 (38%) of the 341 patients, including N1 disease in 97 (28%) and N2 disease in 36 (11%). Of the 44 patients who received chemo alone, 18 (41%) had N1 disease as compared to 28 (26%) of the 107 patients who received chemoXRT and 51 (27%) of the 190 who received chemotherapy and chemoXRT. Patients who received chemotherapy and chemoXRT had the lowest rates of N2 disease (8%, *n* = 15/190) as compared to patients who received chemoXRT (13%, *n* = 14/107) or chemo alone (16%, *n* = 7/44) (*p* = 0.07). In a multivariable logistic regression, receipt of neoadjuvant chemoradiation was associated with a 57% decreased odds (OR: 0.43, 95% CI: 0.23–0.84, *p* = 0.01) of having lymph node positive disease as compared to chemo alone. The median OS by nodal stage was 46 months (N0), 31 months (N1), and 25 months (N2), respectively (*p* < 0.001, Figure 3).

### Impact of Histopathologic Response and Nodal Status on Survival

The presence of lymph node metastases was inversely related to histologic response at the primary tumor (*p* < 0.001). Patients who had a CR, by definition, had no evidence of cancer cells in both the primary tumor and all local-regional lymph nodes. Among the 59 patients with a nCR, 6 (10%) patients had N1 disease, and 3 (8%) had N2 disease. Of the 188 patients with a PR, 65 (35%) patients had N1 disease and 15 (8%) had N2 disease. Finally, of the 79 patients with NR, 26 (33%) had N1 disease, and 18 (23%) had N2 disease. Histologic response in the primary tumor was associated with a lower incidence of lymph nodal metastases (*p* < 0.001).

**Figure 4:**
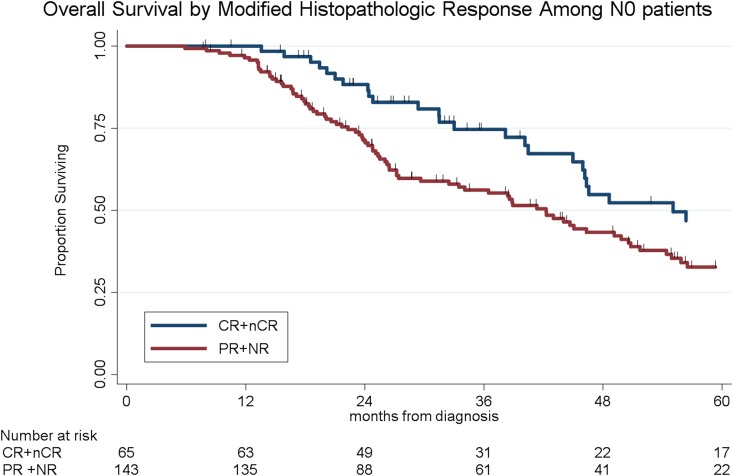
Overall survival by modified histopathologic response among N0 patients.

**Figure 5:**
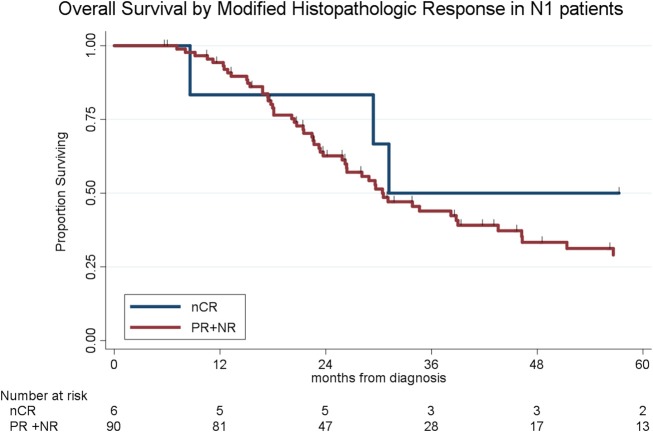
Overall survival by modified histopathologic response among N1 patients.

**Figure 6:**
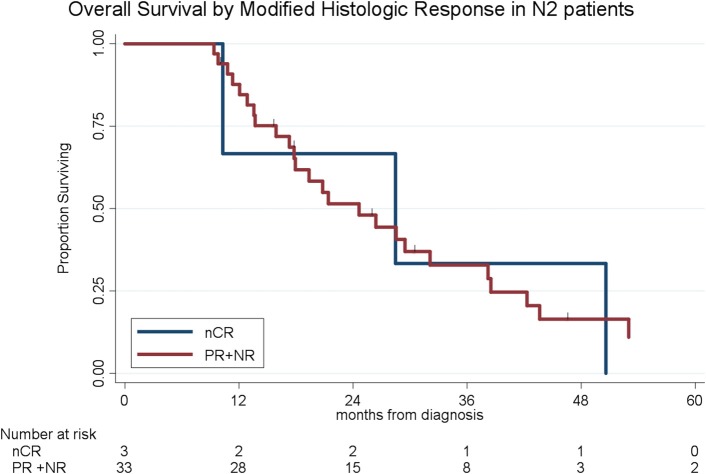
Overall survival by modified histopathologic response among N2 patients.

To determine the impact of histologic response and nodal metastases on OS, we compared the survival by histologic response category among patients with similar nodal status. Among the 208 patients with N0 disease, the median OS of the 65 patients with CR/nCR was 55 months as compared to 42 months for the 143 patients with PR/NR (*p* = 0.02, Figure 4). Among the 96 patients with N1 disease, the median OS of the 6 patients with nCR and the 90 patients with PR/NR was 31 and 30 months, respectively (*p* = 0.28, Figure 5). Of the 36 patients with N2 disease, the median OS of the 3 patients with nCR and the 33 patients with PR/NR was 28 and 24 months, respectively (*p* = 0.89, Figure 6). In an adjusted hazards model, negative prognostic pathologic features included PR/NR (HR: 1.71, 95% CI: 1.15–2.53, *p* = 0.008), N1 disease (HR:1.43, 95% CI: 1.02–2.00, *p* = 0.04), and N2 disease (HR: 2.54, 95% CI: 1.64–3.93, *p* < 0.001), while receipt of adjuvant therapy was protective (HR: 0.62, 95% CI: 0.46–0.83, *p* = 0.001, Table 2).

**Table 2:** Proportional hazards model for overall survival.

	**Univariable**			**Multivariable**		
**Covariate**	**HR**	**95% CI**	***p*-value**	**HR**	**95% CI**	***p*-value**
Modified Ryan Score (ref: CR + nCR)	1.81	1.24–2.62	0.002	1.71	1.15–2.54	0.008
Margin Status (ref: no)	1.44	0.98-2.14	0.06	1.13	0.75–1.69	0.55
Node positive Pathology (ref: N0)	Ref	–	–	Ref	–	–
N1	1.14	1.02–1.94	0.03	1.42	1.02–2.01	0.04
N2	2.50	1.66–3.79	<0.001	2.54	1.64–3.93	<0.001
Any Adjuvant Therapy (ref: no)	0.77	0.58–1.02	0.07	0.62	0.46–0.83	0.001

## Discussion

The purpose of the current study was to evaluate the prognostic importance of treatment response at both the primary tumor and local-regional lymph nodes following neoadjuvant therapy for PC. With regard to the primary tumor, the results of our study corroborate prior findings that a CR following neoadjuvant therapy is uncommon (4%), regardless of the type of neoadjuvant therapy delivered (12, 13, 22). We also observed a superior median OS among patients who had a CR or nCR (median OS = 51 months), as compared to patients with either PR or NR (median OS = 34 months). Unfortunately, a CR or nCR was observed in only 21% of all patients. No differences were observed in the rates of CR or nCR based on the type of neoadjuvant treatment. With regard to lymph node metastases, overall, 61% of patients had N0 disease following neoadjuvant therapy. Furthermore, neoadjuvant chemoradiation was associated with a 57% decreased odds of lymph node metastases as compared to chemo alone. The pathologic response in local-regional lymph nodes was the strongest prognostic factor for OS. Patients with N0 disease experienced an improved median OS (46 months) compared to patients with N1 (31 months) or N2 (25 months) disease. To our knowledge, we are the first to compare the prognostic importance of lymph node metastases combined with an examination of histologic response in the primary tumor following neoadjuvant therapy. We observed that histologic response in the primary tumor was prognostic only among patients with N0 disease. Among patients with N1 or N2 disease, the degree of histopathologic response in the primary tumor did not impact survival. This suggests that the most important prognostic factor following the receipt of neoadjuvant therapy and surgery for operable PC is the status of local-regional lymph nodes.

With the development of recent multidrug regimens for PC, there has been a focus on the ability to achieve a pathologic CR following neoadjuvant therapy and a CR has been considered a surrogate endpoint for clinical trials (23). Rates of CR following neoadjuvant therapy have been reported to range from 3 to 25% (13, 23–26). In the current study, we observed a CR rate of 4% and rates did not differ based on the type of neoadjuvant therapy delivered. Our findings are very similar to rates of CR reported by the largest study to examine pathologic response after neoadjuvant therapy (3.9%) as well as other contemporary studies utilizing neoadjuvant 5-flourouracil, oxaliplatin, and irinotecan (FOLFIRINOX) or gemcitabine/nab-paclitaxelas induction therapy (range 1–4%) (26–28). A unique finding of this study is the observation that the prognostic value of response in the primary tumor must be considered in the context of the status of locoregional lymph nodes. We observed that when persistent disease exists in adjacent lymph node basins following neoadjuvant therapy, the presence of lymph node metastases supersedes any prognostic benefit of treatment response observed in the primary tumor. Whether treatment response in the primary tumor is a meaningful oncologic endpoint remains to be determined. It is important to note that distant recurrence can occur in up to 17% of patients with CRs (26). In this report, 18% of patients with a nCr in the primary tumor had lymph node metastases.

The chemoradiation used in the current study was delivered as intensity modulated radiation therapy (IMRT) and our findings are consistent with reports from other centers which utilized IMRT and reported rates of pathologic CR ranging from 2 to 6% (13, 25, 29). It is important to note that differences in radiation technique may influence the histopathologic response observed in the primary tumor and regional lymph nodes. For example, stereotactic body radiation therapy (SBRT) has been increasingly utilized in the neoadjuvant setting and is given in reduced fractionation, without sensitizing chemotherapy. Such SBRT treatment approaches may or may not include limited radiation to peritumoral lymph nodes. SBRT has been associated with higher rates of pathologic CR in the primary tumor but the effect on lymph node status is unclear. Prior studies of SBRT have omitted the reporting of lymph node metastases and in the limited instances when lymph node status has been reported, node positive disease was as high as 50% (24, 30, 31). In the current study, we observed that histologic response in the primary tumor was of prognostic importance only in patients with node negative disease. Since the technique of SBRT often excludes adjacent lymph nodes, it remains to be seen whether response in the primary tumor will be of prognostic value if patients have residual lymph node metastases. This and other unmet questions may be answered in our currently active randomized controlled trial comparing SBRT vs. conventionally fractionated chemo XRT following neoadjuvant therapy (NCT 03704662).

There is considerable controversy regarding the use of neoadjuvant radiation for PC. Moreover, the optimal method of delivering neoadjuvant radiation and the influence of this therapy on treatment outcomes remains very poorly studied. Since PC is widely believed to be a systemic disease at the time of diagnosis for most patients, the utility of a locoregional treatment modality, such as radiation, seems paradoxical (4). However, proponents of neoadjuvant chemoradiation often argue that patients with PC are at high risk for positive margins (40–60%), tumor-associated periarterial neural infiltration (90%), and lymph node metastases (78%). Not surprisingly, up to 47% of patients with PC will develop local recurrence following a surgery-first approach (5, 32, 33). Unfortunately, local disease recurrence is the sole site of disease recurrence in ~25% patients; could such patients be long term survivors with a more comprehensive operation or the addition of chemoradiation? (6). As systemic therapies continue to improve and potentially decrease early systemic recurrence, the number of patients at risk for local recurrence will increase. The addition of neoadjuvant chemoradiation clearly reduces the rates of positive margins (13%), perineural invasion (60%), and lymph node metastases (40%) compared to a surgery-first treatment approach (27, 34–36). In the current study, we also observed lymph node metastases to be present in 39% of patients, when including both N1 (28%) and N2 (11%) disease. We have previously reported that the addition of neoadjuvant chemoradiation significantly reduced the odds of local-only recurrence (OR: 0.21; 95% CI: 0.06–0.77; *p* = 0.02) (Barnes et al., under review). However, until local-regional recurrence affects a greater percentage of at-risk patients, it will be difficult to demonstrate a statistically significant impact of any local therapy on OS for a disease with such a dominant pattern of distant failure. This does not mean that local therapy has no impact on morbidity and mortality for patients with operable PC—an often-misunderstood concept.

Following the Halstedian principle of tumor progression, if metastatic disease is hypothesized to disseminate through a predictable pattern from local to regional to systemic access, it is not surprising that lymph node metastases are present in up to 78% of patients with PC who undergo immediate surgery (5). Not only the presence of, but the number of lymph node metastases has been associated with OS, as first described by Basturk et al. leading to the revision of lymph node staging to include N1 and N2 disease categories in the 8th edition of the AJCC Staging Manual (37, 38). With a surgery-first approach, the median OS for patients with N0, N1, and N2 disease has been reported as 35, 21, and 18 months, respectively (*p* = 0.004) (37). Following neoadjuvant therapy, lymph node status remains a highly prognostic marker of OS (13, 36, 39, 40). Importantly, lymph node metastases can be sterilized with neoadjuvant therapy, which has been associated with up to a 50% reduction in the frequency of LN positive disease (12, 27, 36). In the current study, patients who were found to have N0 disease had a significant survival advantage (median OS: 46 months) as compared to patients with N1 (median OS: 31months) or N2 disease (median OS: 25 months) (*p* < 0.001). The high rates of N0 disease observed in this study were likely attributable to the delivery of neoadjuvant radiation, as the rates of N0 disease were no different between patients who received chemoradiation alone (61%) and induction chemotherapy followed by chemoradiation (65%) but were superior to patients who received chemo alone (43%, *p* = 0.03). This suggests that the addition of chemoradiation resulted in a stage-migration from node-positive to N0 disease and this was associated with an improvement in OS. Such significant differences in OS, reported across multiple series, presents highly compelling data for the use of pathologic nodal status as an endpoint for phase II clinical trials in operable PC.

In the current study, the hypothesis that improved OS of patients with PC, presumably due to metastatic disease control may be attributable to locoregional therapy (chemoradiation) is unconventional and not generally observed in patients with other solid tumors who receive neoadjuvant therapy. The mechanism for this remains unclear but may be related to two unique biologic characteristics of PC. First, in PC the primary tumor is characterized by the presence of an abundant desmoplastic stroma which acts as a physical barrier to restrict intratumoral drug delivery, creates a hypoxic environment that reduces the efficacy of radiotherapy, and induces an immunosuppressive microenvironment which prevents robust immune infiltration. As such, it is not surprising that treatment response (CR/nCR) in the primary tumor is uncommon, regardless of the type of neoadjuvant therapy delivered. However, PC metastases lose the characteristic stromal component and may even be difficult to distinguish from liver, lung, or ovarian neoplasms (41, 42). In a well-vascularized environment which is depleted of the immunosuppressive stroma, PC metastases may become susceptible to both chemotherapeutic agents as well as chemoradiation. Therefore, it is not surprising that both neoadjuvant chemotherapy and chemoradiation are associated with an increase in lymph node negative disease when compared to patients treated with surgery-first (27, 36, 39). Furthermore, local irradiation of the primary tumor may have immunomodulatory effects (43). Recent studies have demonstrated that radiation induced cell death enhances cross-presentation of antigen and may induce effector T cell responses (44, 45). Therefore, the survival benefit observed in patients with pathologic N0 disease after neoadjuvant chemoradiation may be due to radiation induced tumor cell death which then primes a subsequent immunologic response.

There are limited clinical trials which have examined the survival benefit of radiotherapy in the setting of an intact primary tumor. The first was a multicentered randomized controlled trial through the Eastern Cooperative Oncology Group (ECOG) which included 74 patients with localized unresectable PC (46). Patients were randomized to receive gemcitabine chemotherapy (1,000 mg/m^2^) or gemcitabine plus radiation (600 mg/m^2^, 1.8 Gy/Fx to a total of 50.4 Gy). The radiation included local/regional lymph nodes adjacent to the gross target volume. The median OS for patients who received chemotherapy vs. chemoradiation was 9.2 months vs. 11.1 months (*p* = 0.02). The second trial was an international randomized controlled trial which included 442 patients with locally advanced PC who underwent dual randomization, first for 4 months of gemcitabine (1,000 mg/m^2^) with or without erlotinib (100 mg/d), and second for continued chemotherapy vs. chemoradiation (54 Gy). Radiation encompassed the gross tumor volume but prophylactic irradiation of regional lymph nodes was not performed. Of the 442 patients, 269 underwent a second randomization, and the median OS from the date of first randomization was 16.5 months in the chemotherapy arm as compared to 15.2 months in the chemoradiation arm (*p* = 0.83). Although the two trials resulted in disparate conclusions regarding the impact of radiation on OS in locally advanced PC, it is notable that the improved OS was observed in the clinical trial which included nodal irradiation. These findings intersect with and support the observations of the current study.

The second unique biologic characteristic of PC is the high prevalence of metastatic disease, even among patients with apparent localized disease (4). Although there are many solid tumors which incorporate neoadjuvant radiation into the treatment paradigm, few have demonstrated that neoadjuvant radiation was associated with improved OS. If the addition of radiotherapy can induce an abscopal effect, the impact would be most pronounced in a disease which is enriched for metastatic disease, rather than in a population of patients at low risk of metastatic disease. Given that patients with operable PC are at the highest risk for distant micrometastatic disease (compared to other early stage solid tumors), the down staging effect of radiation may not only impact local regional control but also induce an effective systemic immunologic response which may affect distant micrometastatic disease in patients with adequate intact immunity.

The limitations of this study are consistent with its retrospective nature. First, the assessment of primary tumor response was performed by various board-certified anatomic pathologists, but no interrater variability was assessed between individuals. In addition, the study includes both patients with resectable and borderline resectable disease. Within the cohort of patients with resectable disease, the number of patients treated with neoadjuvant chemotherapy alone is small and our observations regarding pathologic response need to be corroborated in a larger study. There was little variability in the management of patients with borderline resectable PC, as it is our opinion that patients with borderline resectable PC are at greater risk for both systemic disease as well as a positive surgical margin. For that reason, our patients with borderline resectable PC were treated with both neoadjuvant chemotherapy and chemoradiation. Interestingly, we did not observe an incremental benefit in pathologic response at the primary tumor or lymph node basins with the addition of chemotherapy as compared to patients who received chemoradiation alone. Although patients with borderline resectable PC were more likely to have advanced disease, as evidenced by larger tumors and slightly higher pretreatment CA19-9 values, there was no association of improved histopathologic response in the primary tumor or in the nodal basins following chemotherapy and chemoradiation compared to patients with resectable disease who received chemoradiation alone.

## Conclusions

Chemoradiation plays an important role in providing patients with optimal survival outcomes following the receipt of neoadjuvant multimodal treatment for operable PC. Chemoradiation was associated with decreased rates of node positive disease as compared to chemotherapy alone and node negative disease was associated with improved OS. Our current phase II clinical trial of SBRT vs. IMRT will provide further information on the impact of radiation therapy on pathologic outcomes and OS.

## Data Availability Statement

The datasets generated for this study are available on request to the corresponding author.

## Ethics Statement

The studies involving human participants were reviewed and approved by Medical College of Wisconsin IRB. Written informed consent for participation was not required for this study in accordance with the national legislation and the institutional requirements.

## Author Contributions

DW and ST: conception design, analysis, and interpretation. MA: acquisition of data and analysis. BE, WH, MR, KC, CC, AK, JE, BG, PR, NK, DE, CB, and NJ: analysis and interpretation.

### Conflict of Interest

The authors declare that the research was conducted in the absence of any commercial or financial relationships that could be construed as a potential conflict of interest.
